# Spin-wave-mediated mutual synchronization and phase tuning in spin Hall nano-oscillators

**DOI:** 10.1038/s41567-024-02728-1

**Published:** 2025-01-08

**Authors:** Akash Kumar, Avinash Kumar Chaurasiya, Victor H. González, Nilamani Behera, Ademir Alemán, Roman Khymyn, Ahmad A. Awad, Johan Åkerman

**Affiliations:** 1 Department of Physics, University of Gothenburg, Gothenburg, Sweden; 2 Research Institute of Electrical Communication, Tohoku University, Sendai, Japan; 3 Center for Science and Innovation in Spintronics, Tohoku University, Sendai, Japan

**Keywords:** Spintronics, Magnetic devices, Electronic and spintronic devices, Spintronics

## Abstract

Spin–orbit torque can drive auto-oscillations of propagating spin-wave modes in nano-constriction spin Hall nano-oscillators. These modes facilitate both long-range coupling and the possibility of controlling their phase, which is a crucial aspect for device application. Here, we demonstrate variable-phase coupling between two nano-constriction spin Hall nano-oscillators and their mutual synchronization driven by propagating spin waves. Using electrical measurements and phase-resolved micro-focused Brillouin light scattering microscopy, we show that the phase of the mutual synchronization can be tuned by modulating the drive current or the applied field. Our micromagnetic simulations explore the phase tunability using voltage gating. Our results advance the capabilities of mutually synchronized spin Hall nano-oscillators and open the possibilities for applications in spin-wave logic-based devices.

## Abstract (web summary)

Phase tuning of propagating spin waves is a crucial step in the development of devices based on magnons, which are the quanta of spin waves. Now, this has been demonstrated in a device comprising two spin Hall nano-oscillators.

## Main

The generation, propagation and control of magnons—the quanta of spin waves (SWs)—allow the long-range transfer^1,2^ and processing of digital and analogue information^3^ and form the basis of magnonics^4,5^ and SW computing^6,7^. The manipulation of the properties of coherent propagating spin waves (PSWs) in nanoscopic devices, such as their amplitude, phase, propagation direction and interference patterns, holds great promise for designing magnonic conduits with unique properties^8,9^. Various emerging applications, including reconfigurable SW logic circuits^10,11^, unconventional computing^12^ and Ising machines^13^, rely on these advances. Various novel mechanisms have been explored to generate and amplify PSWs^14,15^, such as current-induced spin-transfer torque^16–18^ and spin–orbit torque^19–23^. Nano-constriction spin Hall nano-oscillators (SHNOs) with perpendicular magnetic anisotropy (PMA)^24,25^ are a particularly promising approach, as they are easy to fabricate^26,27^, CMOS compatible^28^, strongly voltage-tunable^29–32^ and known for their superior mutual synchronization at various length scales and dimensions^33–35^.

The mutual synchronization of spin-transfer-torque- and spin–orbit-torque-driven spintronic oscillators is primarily driven by four mechanisms: (1) dipolar coupling^36,37^, (2) direct exchange^38^, (3) electrical current^39,40^ and (4) PSWs^18,38,41–43^. Dipolar coupling and direct exchange decay rapidly with distance^36^. Although an electrical current can couple oscillators over macroscopic distances, it requires magnetic-tunnel-junction-based oscillators with the highest possible magnetoresistance^39,40^. In contrast, PSWs can drive mutual synchronization over micrometre distances independent of magnetoresistance^38,44^. Combining the long-range mutual synchronization of PSWs with the precise control of their frequency, amplitude and phase will be of great importance for emerging SW computing platforms, such as SW Ising machines^13,45^.

Here, we report the experimental observation of variable-phase mutual synchronization of nano-constriction SHNOs. The PSWs locally generated by two oscillators separated by >200 nm allow radiative locking due to in-phase and out-of-phase coupling of the PSWs. This can be further controlled by the electrical current as well as the magnetic field and its orientation. These results were corroborated using state-of-the-art phase-resolved micro-focused Brillouin light scattering (μ-BLS) spectroscopy and micromagnetic simulations. The demonstrated control and manipulation of the relative phase of mutually synchronized oscillators at nanoscopic dimensions holds great promise for various applications such as Ising machines, neuromorphics and SW computing^7,46^.

## Results

### Device fabrication

**Fig. 1:**
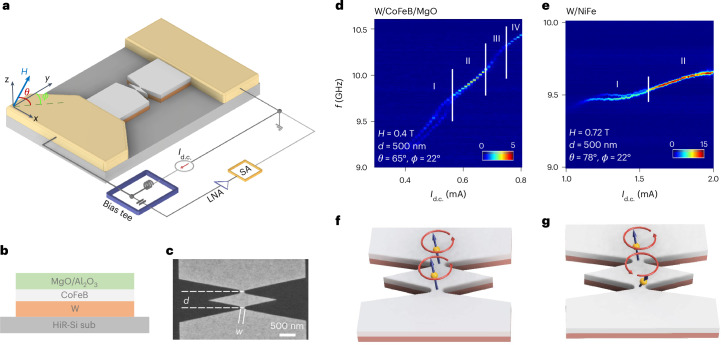
Device fabrication and electrical observation. **a**, Schematic of two nano-constriction SHNOs and their connection to the measurement set-up. Shown is the magnetic field (*H*) and its orientation (*θ* and *ϕ*). **b**, Configuration of the material stack used in the fabrication of the W/CoFeB/MgO SHNOs with PSWs. **c**, Scanning electron microscope image of the fabricated device with dimensions *w* = 150 nm and *d* = 500 nm. **d**,**e**, Power spectral density (PSD) versus applied current (*I*_d.c._) for the nano-constrictions of PMA-based W/CoFeB/MgO (**d**) and in-plane-anisotropy-based W/NiFe (**e**). **f**,**g**, Illustrations of in-phase (**f**) and anti-phase (**g**) mutual synchronization. HiR-Si sub, high-resistivity Si substrate; SA, spectrum analyser; LNA, low-noise amplifier; f, frequency.

Figure 1a shows a schematic of the double-nano-constriction SHNOs and the electrical measurement set-up. To generate PSWs, we used W/CoFeB/MgO trilayers (Fig. 1b), which offer both interfacial PMA^47^ due to the CoFeB/MgO interface and a high spin Hall angle from the W thin film^25,48^. Figure 1c shows a scanning electron microscope image of the fabricated device (width *w* = 150 nm and separation *d* = 500 nm). We fabricated devices with *d* = 200–600 nm to vary the PSW coupling. As control samples without PSWs, we also fabricated W/NiFe-based SHNOs, where the synchronization was driven by dipolar coupling and direct exchange^33^. Further details are given in Methods.

### Electrical observation of synchronization

The PMA raised the auto-oscillating frequency in the nano-constriction above the ferromagnetic resonance (FMR) SW gap of the magnetic layer. This avoided SW localization and, instead, promoted the generation of PSWs^25^. The positive and constant nonlinearity resulted in a linearly increasing auto-oscillation frequency as a function of current, corresponding to SWs with an increasing wavevector (and shorter wavelength). Figure 1d confirms this quasi-linear current dependence of the auto-oscillation frequency in the W/CoFeB/MgO device (*w* = 150 nm and *d* = 500 nm), which had a threshold current (*I*_th_) of just below 0.4 mA, an auto-oscillation frequency of about 10 GHz in a 0.4 T field and about a 15% increase in frequency when the current was increased to 2*I*_th_. In comparison, the W/NiFe device (Fig. 1e; also *w* = 150 nm and *d* = 500 nm but without PMA) had a threshold current of about 1.1 mA, needed a field of 0.72 T to reach about the same frequency (no contribution to effective magnetic field, *H*_eff_, from the anisotropy field) and started with a very weak negative nonlinearity that changed to a weak positive nonlinearity such that the frequency increase was less than 2% at about 2*I*_th_. These very different behaviours are consistent with the W/CoFeB/MgO device generating PSWs and the auto-oscillations of the W/NiFe device being localized.

The presence or absence of PSWs led to different types of mutually synchronized states. The W/CoFeB/MgO nano-constrictions started out in an unsynchronized state (region I), were synchronized between 0.55 and 0.68 mA (region II), showed almost no signal between 0.68 and 0.76 mA (region III), and seemed to synchronize again above 0.76 mA (region IV). By contrast, the W/NiFe nano-constrictions without PSWs exhibited only regions I and II. Although the high-power signal in region II resulted from constructive coherent in-phase interference of the microwave voltage signals from the two mutually synchronized nano-constrictions, corresponding to the state depicted in Fig. 1f, region III represents a type of behaviour consistent with a possible anti-phase mutually synchronized state, as depicted in Fig. 1g. The absence of a microwave signal could, in principle, also indicate so-called oscillation death, which has recently been suggested, occurs in pairs of interacting magnetic-tunnel-junction-based spin-torque nano-oscillators^49–51^. However, a faint residue of a single microwave signal can still be observed in region III, which rules out oscillation death and is, instead, consistent with an out-of-phase, but not strictly anti-phase, mutually synchronized state. Note that region III and the suggested out-of-phase mutually synchronized state were observed only when PSWs were present. In the W/NiFe device, the mutually synchronized state was robust and showed very high output power, consistent with dipolar coupling or direct exchange being responsible for the coupling, both of which favour in-phase mutual synchronization.

### μ-BLS microscopy of the individual nano-constrictions

**Fig. 2:**
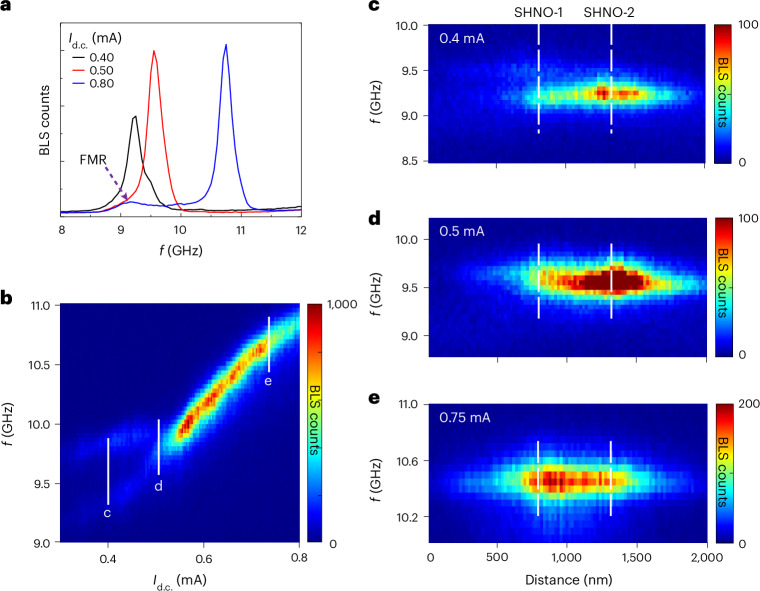
Spatial mapping using μ-BLS. **a**, Representative BLS spectra showing FMR and the auto-oscillations measured at *I*_d.c._ = 0.40, 0.50 and 0.80 mA. **b**, Current-dependent auto-oscillation signal measured using μ-BLS. The solid vertical lines refer to the current values at which BLS spatial maps (**c**–**e**) are taken. **c**–**e**, SW intensity profiles of the double SHNOs along the constrictions, measured at applied current *I*_d.c._ = 0.4 mA (**c**), 0.5 mA (**d**) and 0.75 mA (**e**). The dashed lines indicate the positions of the constrictions. Source data

To conclusively rule out oscillation death and directly visualize the auto-oscillations in each nano-constriction, we present results from μ-BLS. We first used conventional μ-BLS microscopy to map out the SW intensity versus both frequency and spatial coordinates in the double nano-constrictions. The magnetic field conditions were the same as in the electrical measurements illustrated in Fig. 1 (see Methods for details). Figure 2a shows the spectral content of the SWs at three different currents, as measured on the bridge connecting the two nano-constrictions (same device as in Fig. 1d). Note how the high-intensity SW auto-oscillations all lie above the weak thermally excited FMR peak at about 9.1 GHz, which confirms their propagating nature. Figure 2b shows the whole current-dependent spectral distribution of the auto-oscillations, displaying both important similarities and differences compared with the electrical data in Fig. 1d. At low currents, we observe two faint signals with about the same threshold as in Fig. 1d. At about 0.55 mA, the two signals merge and the counts for Brillouin light scattering (BLS) increase strongly and remained high for all higher currents. As in the electrical measurements, the frequency dependence was essentially linear in current, consistent with PSWs above FMR.

It is straightforward to identify the behaviour below 0.55 mA as being due to two unsynchronized nano-constrictions and, hence, identical to region I of the electrical data. However, above 0.55 mA, the situation was different. Although it is again straightforward to identify the state above 0.55 mA with two mutually synchronized nano-constrictions (region II), there was no sign of any transition at 0.68 mA into a region III with strongly decreasing BLS counts. Instead, the BLS counts remained essentially constant across 0.68 mA and continued to display all the characteristics of a mutually synchronized state with high SW intensity. This rules out the possibility of oscillation death being the reason for the very weak electrical signal in region III.

To gain further insight into the auto-oscillation modes, we present in Fig. 2c–e hybrid frequency–spatial BLS maps for a few selected *I*_d.c._ along a line through the double nano-constrictions. At *I*_d.c._ = 0.4 mA, the spatial maps indicate an unsynchronized state, with SHNO-1 having a higher frequency but lower counts than SHNO-2 (there was substantial leakage of the SHNO-2 signal into the SHNO-1 region due to the 300 nm laser spot size; this should not be interpreted as SHNO-1 auto-oscillating on this frequency). At *I*_d.c._ = 0.5 mA, the two oscillators were close to being, but not yet, synchronized. SHNO-1 now had higher counts and its frequency had been pulled closer to that of SHNO-2. The BLS map remained asymmetric about its central frequency, indicating that the two regions were not yet mutually synchronized. However, at *I*_d.c._ = 0.75 mA, which was well inside region III, the BLS map was symmetric across its central frequency and both SHNOs showed higher counts, indicative of a mutually synchronized high-intensity state. Again, this rules out oscillation death and corroborates out-of-phase mutual synchronization as the probable explanation. To directly measure the internal relative phase of the mutually synchronized state, we, therefore, resorted to phase-resolved μ-BLS microscopy.

### Direct observation of phase using phase-resolved μ-BLS microscopy

As described in Methods, phase-resolved μ-BLS microscopy was used to determine the phase of the detected SWs with respect to a reference signal. This is usually done by exciting the SWs directly with the reference signal fed to an antenna, for example^52^. However, in SHNOs, the SWs are generated intrinsically by auto-oscillations, so to study their phase, one must first injection-lock the SHNO to the reference signal^45^. Strictly speaking, phase-resolved μ-BLS microscopy does not study the free-running nano-constriction pair, only the corresponding injection-locked system, which may or may not resemble the free-running mutually synchronized state. We minimized the injection-locking signal power to maintain a stable locked state while keeping perturbations low, aiming to extract an oscillation phase close to the free-running case.

**Fig. 3:**
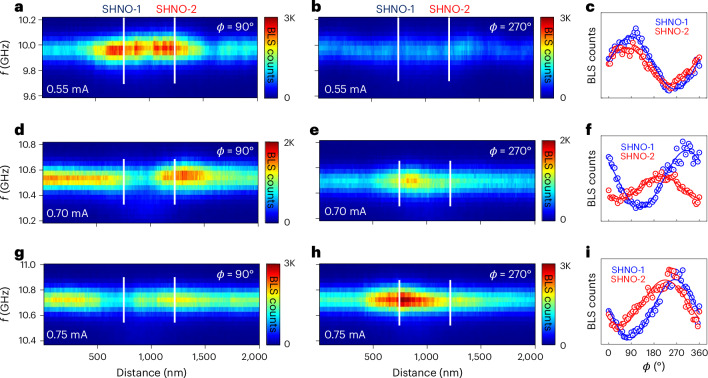
Phase-resolved μ-BLS measurements. **a**,**b**,**d**,**e**,**g**,**h**, Phase-resolved SW intensity maps of the double SHNOs measured with two different phase settings (*ϕ*) separated by 180°. **a**, *I*_d.c._ = 0.55 mA, *ϕ* = 90°. **b**, *I*_d.c._ = 0.55 mA, *ϕ* = 270°. **d**, *I*_d.c._ = 0.70 mA, *ϕ* = 90°. **e**, *I*_d.c._ = 0.70 mA, *ϕ* = 270°. **g**, *I*_d.c._ = 0.75 mA, *ϕ* = 90°. **h**, *I*_d.c._ = 0.75 mA, *ϕ* = 270°. **c**,**f**,**i**, BLS counts as a function of *ϕ* measured at the centre of each nano-constriction for *I*_d.c._ = 0.55 mA (**c**), *I*_d.c._ = 0.70 mA (**f**) and *I*_d.c._ = 0.75 mA (**i**). The symbols are the measured counts at an injection of *P*_IL_ = 10 dBm. the solid lines are sinusoidal fits. Source data

Figure 3 shows the phase-resolved μ-BLS results for the W/CoFeB/MgO device at three different current levels: *I*_d.c._ = 0.55 mA (region II) and *I*_d.c._ = 0.70 and 0.75 mA (region III). At all times, the device was injection-locked with minimal power *P*_IL_ = −10 dBm and *f*_IL_ = *f*_SHNO_. Figure 3a shows a hybrid frequency–spatial map of the phase versus BLS counts as a function of frequency and position along the line connecting the two nano-constrictions. The phase angle with respect to the reference was set to *ϕ* = 90° (controlled by an electrical phase shifter). Figure 3b shows the corresponding counts when the phase shifter was rotated to *ϕ* = 270°. It is evident from these two plots that the two nano-constrictions were in phase with each other and contributed about equal counts to the BLS intensity. Figure 3c shows the full phase-dependent BLS counts extracted from the locations of the two nano-constrictions (vertical white lines in Fig. 3a,b) when *ϕ* was varied from 0° to 360°. Sinusoidal fits to the experimental data yielded a small relative phase difference of Δ*ϕ* = 17 ± 3° between the two SHNOs. The phase-resolved μ-BLS results, hence, corroborate the conclusion from the electrical measurements that the two nano-constrictions largely auto-oscillated in phase.

However, the situation is dramatically different in region III. Figure 3d–i shows the corresponding phase-dependent results at *I*_d.c._ = 0.70 and 0.75 mA. The two nano-constrictions show very different behaviour in the BLS maps. When the full phase-dependent counts were fitted, we extracted very large relative phases of Δ*ϕ* = 100 ± 5° at 0.70 mA and Δ*ϕ* = 51 ± 5° at 0.75 mA. As already indicated by the electrical data, region III is, hence, conclusively characterized by out-of-phase mutual synchronization, which explains the almost vanishing electrical signal in this region. Similar results for a separation of *d* = 700 nm are shown in Supplementary Figs. 7 and 8.

### Micromagnetic simulations

To corroborate our experimental findings, we carried out micromagnetic simulations using MuMax3 (ref. ^53^) to reproduce and further understand the experimentally observed behaviour. The simulated device was identical to the W/CoFeB/MgO double SHNO with two 150-nm-wide nano-constrictions with 500 nm separation. The magneto-dynamical parameters for the simulations were extracted from the experimental data obtained through spin-transfer FMR measurements of W/CoFeB/MgO microstrip devices^35,48^. A detailed description of the simulations is presented in Methods.

**Fig. 4:**
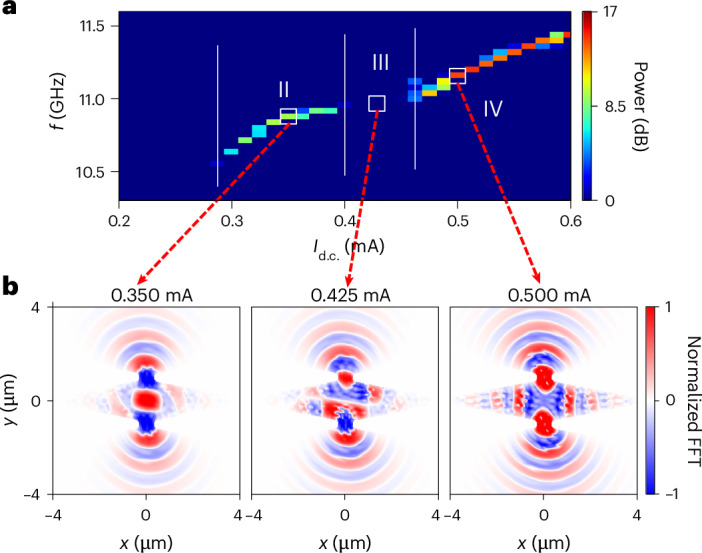
Micromagnetic simulations. **a**, Simulated PSD versus *I*_d.c._ for two 150-nm-wide nano-constriction SHNOs separated by 500 nm, which reproduces the three mutually synchronized regions (II–IV) observed in the electrical measurements. **b**, Complex auto-oscillation mode profiles for the current in each region. The shape of the resonant modes in the bridge connecting the constriction influences their stationary phase convergence. FFT, fast Fourier transform.

Figure 4a shows the simulated power spectral density (PSD) as a function of the applied direct current (d.c.), which reproduces the experimental results very well with minor expected differences: (1) The threshold current in the micromagnetic simulations (*T* = 0 K) was slightly lower than in the room-temperature experiments. (2) The two identical nano-constrictions were already synchronized at the threshold as they auto-oscillated at exactly the same frequency. That is, there was no region I. Except for this region, we identified the same behaviours shown in Fig. 1d. Region II is a high-power in-phase mutually synchronized state. In region III, the microwave signal disappeared, and the region corresponds to an anti-phase mutually synchronized state. In region IV, a strong microwave signal reappeared, and the region corresponds to in-phase mutual synchronization. Figure 4b shows spatial maps of the complex Fourier transform at the corresponding auto-oscillating frequencies in regions II–IV. The phase of the SWs confirms the in-phase mutual synchronization in regions II and IV and the anti-phase mutual synchronization in the middle of region III. We, thus, discarded oscillator death as a possible explanation for this behaviour, as also shown in Supplementary Fig. 9a and Supplementary Video 1.

### Current-controlled variable-phase mutual synchronization

**Fig. 5:**
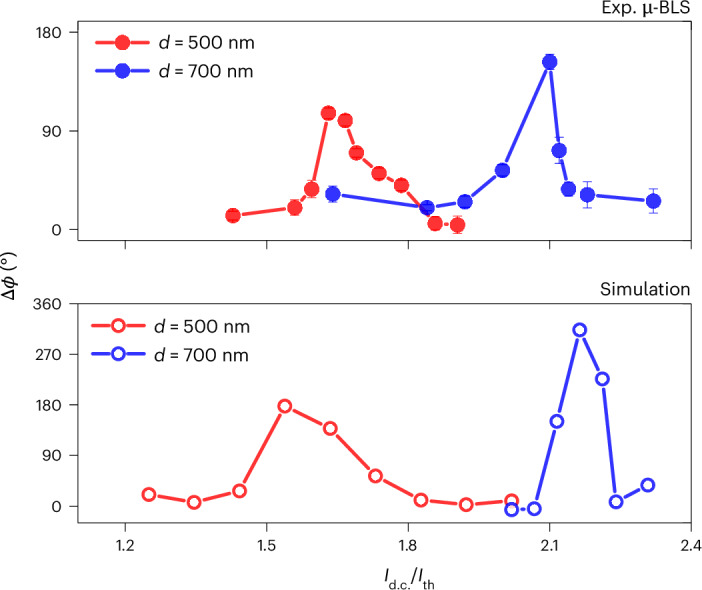
Evolution of the relative phase. The variable phase difference Δ*ϕ* between two mutually synchronized SHNOs (at *d* = 500 or 700 nm) as a function of criticality (*I*_d.c._/*I*_th_) obtained from phase-resolved μ-BLS experiments (top) and micromagnetic simulations (bottom). Exp., experimental. Source data

We next demonstrate how the drive current can continuously tune the internal phase of the mutually synchronized state and, hence, the coupling phase. Figure 5 shows the experimental dependence of the internal relative phase difference Δ*ϕ* on the current for the mutually synchronized state, as extracted from phase-resolved μ-BLS measurements on W/CoFeB/MgO SHNO pairs with *d* = 500 or 700 nm. The internal phase was essentially zero at low current, increased to a maximum (106° for *d* = 500 nm and 153° for *d* = 700 nm) at intermediate current values and then decreased back towards zero at the highest currents.

The micromagnetic simulations in Fig. 5 reproduce the experimentally observed phase tuning very well, albeit with slightly different current values and a higher peak value of the phase difference. We ascribe the difference in peak height to the aforementioned limitation of phase-resolved μ-BLS microscopy, as it requires the devices to be injection-locked to extract the phase. Although the micromagnetic simulations can extract the true Δ*ϕ* of the simulated SHNOs, the injection-locking signal will reduce the experimental Δ*ϕ* when it interacts with the oscillators, as the injection-locking signal will try to align both their phases with its own. This can be demonstrated experimentally by increasing the strength of the injection-locking signal beyond the minimum value for injection-locking. A plot of Δ*ϕ* versus *P*_IL_ (Supplementary Fig. 6) shows how the extracted relative phase of the *d* = 500 nm device decreased from 100° to 60° with increasing injected power. It is reasonable to expect that this trend will continue if a lower power is used. Although the intrinsic, unperturbed value is, therefore, out of reach, the overall trends of the experiment and the simulation in Fig. 5 agree very well.

Spin-transfer-torque-generated PSWs were first studied by Slonczewski^54^, and although his derivation concerns nano-contact spin-torque nano-oscillators, the same principles apply to the spin–orbit torque-driven generation of PSWs in nano-constriction SHNOs^25^. When the drive current exceeds a certain auto-oscillation threshold, increasing the current further increases the auto-oscillation amplitude. The nonlinearity of the system then determines how the operating frequency responds to this amplitude increase^38^. In our case, the positive nonlinearity increased the frequency as the current was increased. The auto-oscillating magnetization in the nano-constriction can couple to available SW modes outside the auto-oscillation region, requiring these SWs to match the auto-oscillation frequency. These SWs are PSWs radiating from the nano-constriction, with a wavelength determined by the frequency through the exchange constant of the magnetic material. Shorter wavelengths correspond to higher PSW frequencies due to the increased exchange energy term in the effective field. When the PSWs dominate the coupling, they want their wave patterns outside the auto-oscillation regions to be in phase. As the nano-constriction locations are fixed, keeping the wave patterns in phase while the wavelength changes can be accomplished only by changing the relative phase of the two auto-oscillations. Consequently, the continuous tuning of the relative phase is a direct consequence of the current-dependent wavelength of the PSWs. Supplementary Fig. 10 shows the PSW wavelength extracted from micromagnetic simulations of the *d* = 500 nm device, demonstrating the expected quasi-linear decrease with increasing drive current.

In addition, to confirm the robustness, repeatability and control of the variable-phase mutual synchronization, we experimentally explored devices with SHNOs at various separations for both W/NiFe and W/CoFeB/MgO nano-constriction pairs. The results are summarized in Supplementary Figs. 1 and 2. By employing narrow bridges to reduce SW damping, we also demonstrated mutual synchronization at larger separations (up to 2 μm) with both in-phase and out-of-phase locking, thus confirming the long-range synchronization of SHNOs while preserving phase information and highlighting the generality of variable-phase synchronization across different SHNO designs.

### Varying the applied magnetic field

Supplementary Fig. 4a–l depicts PSD versus d.c., measured for field strengths ranging from 0.35 to 0.46 T with a step size of 0.01 T for W/CoFeB/MgO double nano-constrictions separated by 420 nm. As expected, both the auto-oscillation frequency and threshold current increased quasi-linearly with field strength. The location of region III also systematically depended on the field strength. Supplementary Fig. 4m summarizes the results. The central frequency of the auto-oscillations was extracted only when the PSD was above a certain value (>0.3 dB over noise). This approximately captured the beginning and the end of region III for all field strengths. Much in the same way as the threshold current increased with field strength, the location of region III shifted quasi-linearly to higher currents with higher fields. To a first approximation, the difference in the current between the location of region III and the threshold current stayed constant with increasing field. This is consistent with the wavevector of the PSWs being independent of the external field strength but directly dependent on the criticality *I*_d.c._/*I*_th_ (see Discussion).

Supplementary Fig. 5 shows the PSD versus d.c. as a function of the out-of-plane angle (*θ* = 55°–68°) for the same double nano-constriction. The dependence on *θ* is considerably more complex than on field strength, with two regions of signal extinction appearing at lower angles. These regions seem to merge at higher angles.

### Voltage control of the phase difference

In large SHNO arrays, we experimentally demonstrated control of the relative phase between mutually synchronized SHNOs by changing the excitation current. For actual applications, this is not particularly useful, as one would like to control the pairwise coupling phase individually. We propose voltage-controlled magnetic anisotropy (VCMA) as an efficient mechanism for controlling the relative phase between SHNO pairs in large arrays. VCMA has been used successfully to control the magnetization dynamics of single SHNOs by modifying their frequency, threshold current and even effective damping^29,32,55^. This localized tuning of the magnetic environment changes the dispersion relation and the related phase and group velocities of PSWs between the constrictions, thus paving the way towards the effective control of the relative phase between SHNOs. Even more, by tuning the applied voltage, one can alter the energy landscape of propagating magnons, thus tuning their transmission properties^55^.

**Fig. 6:**
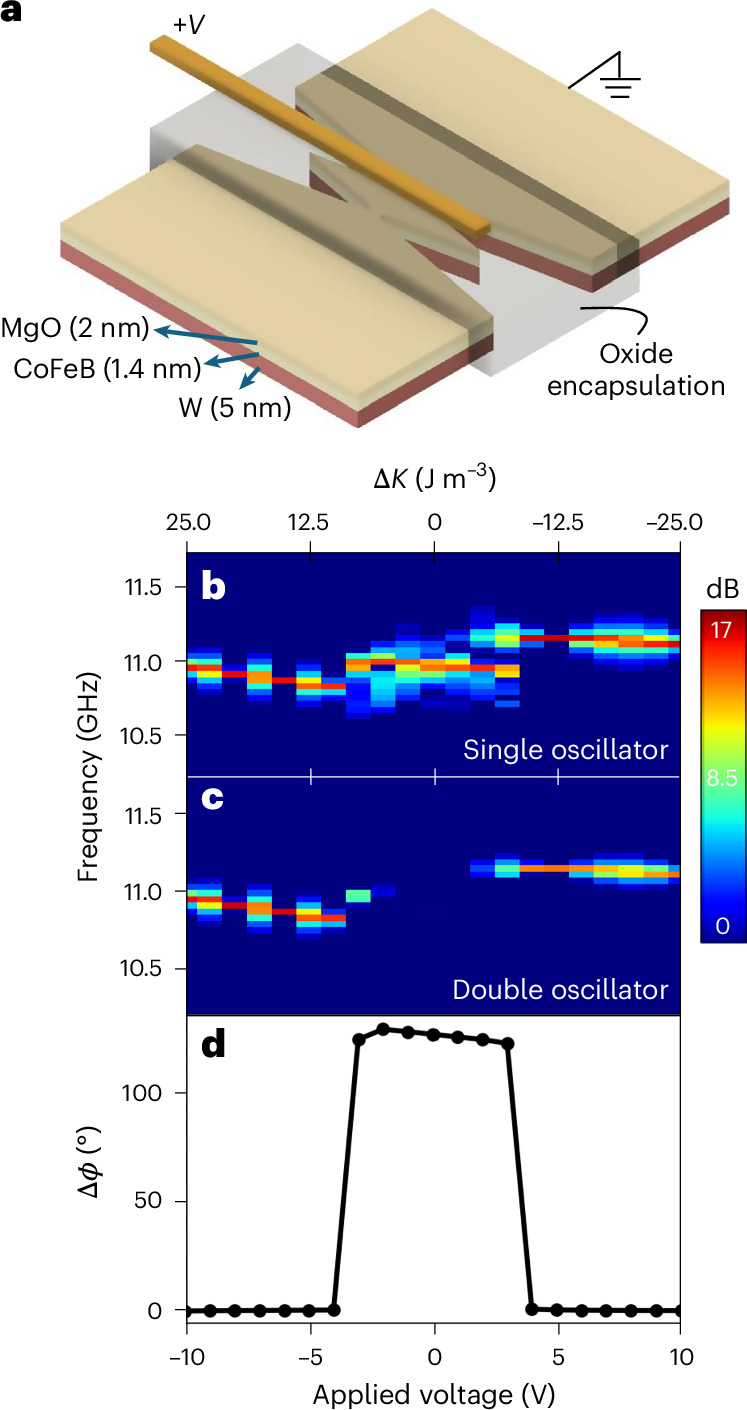
Effects of voltage gating on the mutual synchronization of SHNOs. **a**, Simulated device. The extra MgO layer induces VCMA on CoFeB by allowing the voltage to modify the interfacial PMA. **b**, PSD of a single oscillator as a function of applied voltage. Notice a discontinuity in the auto-oscillation frequency but no oscillator death. **c**, PSD of a double oscillator as a function of applied voltage. The gap in the middle shows a lack of phase synchronization between the two SHNOs. **d**, Phase difference as a function of applied voltage. It takes as little as ±4 V to make the SHNOs in phase. Source data

Figure 6a shows a schematic of the simulated device. VCMA was induced by adding an MgO layer and a rectangular 150-nm-wide gold electrode. The interfacial PMA between the oxide and ferromagnetic layer is tuned by the voltage applied on the electrode, which creates a rectangular VCMA gate on the bridge that can control the PSW dispersion and, thus, the phase locking of the SHNOs.

Figure 6b,c shows the results of the simulation as a function of applied voltage for single (Fig. 6b) and double (Fig. 6c) oscillators for a current *I*_d.c._ = 0.425 mA, the current with the largest relative phase. Applying a gate voltage created either a potential wall (negative voltage) or well (positive voltage) for the PSWs. We found that voltages higher than ±4 V switched the coupling phase to zero (Fig. 6d), which led to the high output signal shown in Fig. 6c. A voltage gate placed between neighbouring SHNOs can, hence, switch the sign of the coupling, which, for example, is sufficient to map MAX-CUT problems onto SHNO-based Ising machines^56^. To achieve continuous voltage tuning of the phase, one probably would have to place the gate differently and possibly asymmetrically. However, such an investigation is beyond the present study.

## Discussion

The strong PMA of the W/CoFeB/MgO material stack counteracts the shape anisotropy and negative nonlinearity of the thin-film geometry and, with the help of a moderate applied field, pulls the magnetization out of plane and turns the nonlinearity positive. This leads to magnon–magnon repulsion and the excitation of PSWs^25^. Thus, the qualitative behaviour of the mutual synchronization of SHNOs can be approximately described using the ordinary SW dispersion of an out-of-plane magnetized film^57^: $f={f}_{{{\rm{FMR}}}}+\frac{\gamma }{2\uppi }D{k}^{2}$, where *f*_FMR_ is the frequency of the FMR, γ is a gyromagnetic ratio, *D* ≃ 2*A*_ex_/*M*_s_ is the dispersion coefficient, defined by the exchange stiffness *A*_ex_ and saturation magnetisation *M*_s_, and *k* is a wavevector of the SW. Our micromagnetic simulations show that the PSW already has a substantial wavevector at the auto-oscillation threshold (Supplementary Fig. 10), as it is mainly defined by the geometry of the constriction. This behaviour is largely identical to the original description of PSWs in nano-contact spin-torque nano-oscillators^54^, where the wavevector at the threshold is given by the nano-contact diameter. With increasing criticality (current increasing beyond the threshold), the wavevector further increases. Considering the frequencies at the start of regions II (*f*_II_ = 9.75 GHz) and III (*f*_III_ = 10.1 GHz; Fig. 1d) and taking *f*_FMR_ = 9.3 GHz (Fig. 2a), we get the wavelengths *λ*_II_ = 304 nm and *λ*_III_ = 228 nm. This is in good agreement with the results of the simulations for PSWs outside the constriction region (Fig. 4). Interestingly, because the applied magnetic field contributes mainly to the first term of the dispersion law, that is to *f*_FMR_, and anti-phase locking occurs at the same wavevector for different field values, region III moves in parallel with the threshold current when the field strength is varied (Supplementary Fig. 4).

Note that the wavelengths obtained do not coincide with the distance between SHNOs as they are related to PSWs outside the nano-constriction and bridge region. Moreover, our experiments did not show a clear dependence of the position of region III on the nano-constriction separation (*d*) (Supplementary Fig. 2). The micromagnetic simulations provide further insight into this behaviour, as they reveal the crucial importance of the SW patterns inside the rhombic bridge connecting the two nano-constrictions. The complex profiles of the SWs within the rhombic bridge are drastically different from those of freely PSWs outside the SHNOs, which highlights the importance of the particular SW modes in this area (Fig. 4b). As a result, the distance between two in-phase oscillating points was very different from the wavelength of a free SW. The dependence on the details of the SW modes of the bridge also explains the complexity of how region III depends on the out-of-plane angle, which has a strong impact on the nonlinearity, with non-trivial consequences for the particular SW modes that are dominant in the bridge.

The sensitivity to the details of the bridge also explains the lack of systematics when we varied *d* (Supplementary Fig. 2). Conversely, this sensitivity should allow for sensitive control of the phase of the mutual synchronization, both through the shape and dimensions of the bridge and, more interestingly, through voltage control of the PMA^29^ in the bridge region. As the PSWs fill up the bridge region, it might be sufficient to fabricate voltage gates on the two sides of the bridge to avoid any detrimental processing damage in the central region between the two nano-constrictions. Although we have focused on rhombic bridges in this study, there is great freedom in future bridge designs with or without voltage gates in different locations. This could lead to very rich variable-phase phenomena in the coupling between adjacent nano-oscillators, with direct applications in neuromorphic computation and Ising machines.

## Methods

### Fabricating SHNOs

Thin-film stacks of W(5 nm)/CoFeB(1.4 nm)/MgO(2 nm)/Al_2_O_3_(4 nm) and W(5 nm)/NiFe(3 nm)/Al_2_O_3_(4 nm) were prepared on a high-resistivity Si substrate (*ρ* > 20,000 Ω cm) using d.c./radio-frequency magnetron sputtering (AJA Orion 8) at room temperature. The W/CoFeB/MgO thin films were annealed post deposition for 1 h at 300° under an ultrahigh vacuum to induce interfacial PMA. The SHNO devices of width 150 nm were fabricated using electron-beam lithography (Raith EBPG 5200) followed by Ar-ion milling^32^. For the mutual synchronization experiments, double nano-constrictions were fabricated with various separations (200–600 nm). The ground–signal–ground contact pads were fabricated in a subsequent step using maskless ultraviolet lithography (Heidelberg Instruments MLA 150) and a lift-off technique. Cu(800 nm)/Pt(20 nm) for the contact pads was deposited by d.c. magnetron sputtering.

### Electrical measurements

The electrical measurements in the characterization of the free-running properties of SHNO devices were performed using a custom-designed ground–signal–ground pico-probe (150 μm pitch, GGB Industries) placed between the electromagnet poles. The motorized sample stage could be rotated, thus allowing us to apply an out-of-plane magnetic field. A d.c. was supplied to the SHNO devices using a constant current source (KE 6221). A magnetic field of 0.4–0.8 T was applied at a 65° out-of-plane angle and a 22° in-plane angle to achieve PSW modes in CoFeB thin films^25,58^. The generated microwave auto-oscillations were amplified using a low-noise amplifier (32 dBm, BnZ Technologies) and observed using a spectrum analyser (R*&*S FSV) with a resolution bandwidth of 1 MHz. All measurements were performed at room temperature.

### Phase-resolved μ-BLS measurements

The magneto-optical measurements were performed using μ-BLS. A monochromatic continuous wave laser (wavelength of 532 nm) was focused on the nano-constriction region by a ×100 microscope objective with a large numerical aperture (0.75) down to the 300 nm diffraction limited spot diameter. The magnetic field conditions were kept almost identical to those used in the electrical measurements. To capture the phase-resolved information, we modulated the phase of the incoming light using an electro-optical modulator operating at the same frequency as the injection signal applied to the double-nano-constriction SHNOs. The basic principle relies on interference between elastically scattered light (a phase controlled light with an electrical phase shifter) and light carrying phase information inelastically scattered from the oscillators^52,59^. The resulting BLS signal was due to interference between both types of scattered light, with the signal being highest when the phase difference between the electro-optically modulated light (reference light) and the oscillator was minimal and small when the interference was destructive, which occurred when the oscillator and the electro-optically modulated reference were out of phase. The resultant signal was analysed with a Sandercock-type six-pass tandem Fabry–Perot interferometer (TFP-1, JRS Scientific Instruments). A three-axis nanometre-resolution stage, along with an active stabilization protocol provided by THATec Innovation, was employed to provide precise long-term spatial stability during the measurement. All measurements were performed at room temperature.

### Micromagnetic simulations

The ferromagnetic layer was simulated micromagnetically using the GPU-accelerated programme MuMax3 (ref. ^53^). The volumetric current density and Oersted field were generated using COMSOL Multiphysics 6.1. The electron-beam lithography schematics used for sample fabrication were imported directly into COMSOL. A bilayer device of W(5 nm)/CoFeB(1.4 nm) was simulated using the Magnetic and Electric Fields (mef) package, with the electrical properties of materials taken directly from our measurements.

The 4 μm × 4 μm × 1.4 nm double-nano-constriction SNHO geometry obtained from the fabrication schematics was discretized into 512 × 512 × 1 cells. The material parameters used in the simulations were either measured directly from the samples using FMR (saturation *M*_S_ = 1,050 kA m^−1^, gyromagnetic ratio *γ*/2π = 29.1 GHz T^−1^, Gilbert damping constant *α* = 0.025 and PMA field *K*_u_ = 645 kJ m^−^^3^) or taken from the literature (*A*_ex_ = 19 × 10^−12^)^60^. This system was excited with d.c. biasing currents between 100 and 600 μA. The out-of-phase spin-polarized currents from the W layer were calculated using the spin Hall angle *θ*_SH_ = 0.6, measured from microstrip devices^48^. The torque generated by this current was calculated for each cell and added using a fixed layer at the bottom of the CoFeB film. The magnetic dynamics of the ferromagnetic film was simulated by integrating the Landau–Lifshitz–Gilbert–Slonczewski equation over 30 ns. We found that the relative phase between constrictions settled after 15 ns for all currents evaluated.

The PSDs were calculated using a complex fast Fourier transform of the time evolution of the average magnetization of the whole sample and each of the constrictions. The mode profiles of the device were obtained using a complex point-wise fast Fourier transform for the full magnetization maps. The phase difference between the nano-constrictions was extracted from line scans of the auto-oscillation mode profiles, as detailed in Supplementary Fig. 6b.

## Online content

Any methods, additional references, Nature Portfolio reporting summaries, source data, extended data, supplementary information, acknowledgements, peer review information; details of author contributions and competing interests; and statements of data and code availability are available at 10.1038/s41567-024-02728-1.

## Supplementary information

Supplementary Information Supplementary Notes 1–3 and Figs 1–10.

Supplementary Video 1 Micromagnetic simulation of PSWs in SHNOs at 425 μA and 500 μA charge current.

## Source data

Source Data Fig. 2 BLS spectra at three current values.

Source Data Fig. 3 Phase relation (experimental data and the fitting curves).

Source Data Fig. 5 Evolution of relative phase (experiments and simulation).

Source Data Fig. 6 Evolution of relative phase versus gate voltage (simulation).

## Data Availability

Source data are provided with this paper and are available via Zenodo at 10.5281/zenodo.13891411 (ref. ^61^). All other data that support the conclusions of this work are available from the corresponding authors upon reasonable request.

## Code availability

The codes used to perform the simulations and to analyse the data in this work are available via Zenodo at 10.5281/zenodo.13891411 (ref. ^61^). The micromagnetic simulations were performed using the MuMax3 programme^53^.
