# Supplementary material for: Spin-wave-mediated mutual synchronization and phase tuning in spin Hall nano-oscillators
Source: Nat Phys. 2025 Jan 8;21(2):245–52. doi: 10.1038/s41567-024-02728-1 (PMC11825361; doi:10.1038/s41567-024-02728-1)
Supplement: Supplementary file 1 — Supplementary Notes 1–3 and Figs 1–10. [file 41567_2024_2728_MOESM1_ESM.pdf]

nature physics

Supplementary information

https://doi.org/10.1038/s41567-024-02728-1

Spin-wave-mediated mutual synchronization and phase tuning in spin Hall nano-oscillators

In the format provided by the authors and unedited

Contents

1 Electrical Measurements **S2**

1.1 Supplementary Note 1. In-plane *vs.* out-of-plane magnetized system S2

1.2 Supplementary Note 2. Separation dependence of variable synchronization S2

1.3 Supplementary Note 3. Variable phase synchronization at large separations S4

1.4 Supplementary Note 4. Magnetic field dependence of variable synchronization S5

1.5 Supplementary Note 5. Magnetic field orientation of variable synchronization S6

2 Micro-focused BLS microscopy **S7**

2.1 Supplementary Note 6. The relative phase difference between two SHNOs measured at I_{DC} = 0.70 mA for different RF power (P_{IL}) S7

2.2 Supplementary Note 7. μ-BLS microscopy of the individual nano-constrictions with *d* = 700 nm . S7

2.3 Supplementary Note 8. *phase-resolved* μ-BLS microscopy of the individual nano-constrictions with *d* = 700 nm S9

3 Micromagnetic simulations **S10**

3.1 Supplementary Note 9. Individual oscillators S10

3.2 Supplementary Note 10. Phase difference between oscillators S11

3.3 Supplementary Note 11. Wave-length of spin waves versus I_{DC} S12

1 Electrical Measurements

1.1 Supplementary Note 1. In-plane *vs.* out-of-plane magnetized system

In addition to the detailed results in the main manuscript, we explored a large number of additional devices under different measurement conditions to confirm the robustness, repeatability, and control of the variable-phase mutual synchronization. As shown in micromagnetic simulations, changing the nano-constriction separation from 500 to 700 nm immediately impacts the phenomenon as different wave vectors match different separations. Figure S1(a-f) compares the power spectral density (PSD) in double nano-constriction spin Hall nano-oscillators (SHNOs) with different widths ($d = 300$ nm, 400 nm, and 500 nm) fabricated using in-plane magnetized W/NiFe (a-c) and perpendicular magnetic anisotropy (PMA)-based W/CoFeB/MgO thin films (d-f). It is evident that, although the W/NiFe system exhibits positive non-linearity, no out-of-phase synchronized modes (Region III, as discussed in the manuscript) are observed for W/NiFe due to the absence of propagating spin wave (PSW) modes. These results underscore the necessity of PSWs to observe such phenomena. The double nano-constriction SHNOs based on W/CoFeB/MgO clearly exhibit Region III (out-of-phase synchronization) for varying current values.

As in Fig. 1e in main manuscript, the W/NiFe pairs consistently show only Region I & II, with Region II starting at increasingly higher currents the greater the separation. This is consistent with the coupling mechanism being dipolar and/or direct exchange, essentially identical to earlier experimental reports (Ref. 33). In contrast, the three W/CoFeB/MgO devices all show regions of disappearing microwave signal (Region III), indicating out-of-phase mutual synchronization.

1.2 Supplementary Note 2. Separation dependence of variable synchronization

According to the previous theoretical predictions, made for circular nano-contacts (Ref. 42), the phase in the locked regime should behave quasiperiodically on the distance d with a periodicity defined by the wavelength λ_{SW} of the emitted spin waves. A similar result was obtained by simulations for the short spin waves in a nano-constriction device (Ref. 43). To investigate whether the location of Region III depends systematically on nano-constriction separation (d), we fabricated a large set of nano-constriction pairs with finer steps in d. PSD measurements of

11 such devices with d ranging from 200 to 560 nm are shown in Fig. S2. While all devices show regions of disappearing microwave signal, the location of these regions does not show a clear trend. One of the possible reasons is the complex 2D pattern of the excited PSW modes on the bridge ((Rhombus)), which changes λ_{SW} into a less well-defined value that no longer scales linearly with the bridge dimensions. Another possible extrinsic reason is the inevitable difference in the physical oscillators due to uncertainties in the fabrication processes. Combined with the complex dependence on the exact 2D patterns of the PSW modes, such fabrication variations may also give complex results. Hence, proposing a clear dependence of Region III with varying d (with respect to current values) is challenging, primarily due to the intricate dynamics in varying bridge (Rhombus) sizes with separation with additional contribution due to uncertainty in nano-fabrication making the SHNOs non-identical.

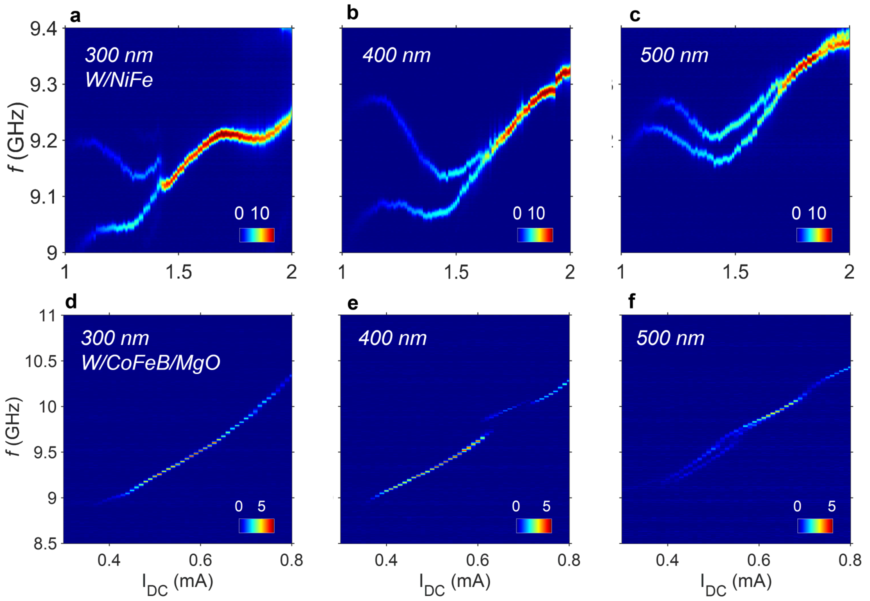

Figure S1: (a-c): PSD *vs.* direct current for W/NiFe thin film nano-constriction SHNO with varying separation (d) between SHNOs (a. 300 nm, b. 400 nm and c. 500 nm, respectively), measured at H = 0.7 T, θ=82° and ϕ=20° angle. (d-f): PSD *vs.* direct current for W/CoFeB/MgO thin film nano-constriction SHNO with varying separation between SHNOs (d: 300 nm, e: 400 nm and f: 500 nm, respectively, measured at H = 0.4 T, θ=65° and ϕ=22° angle.

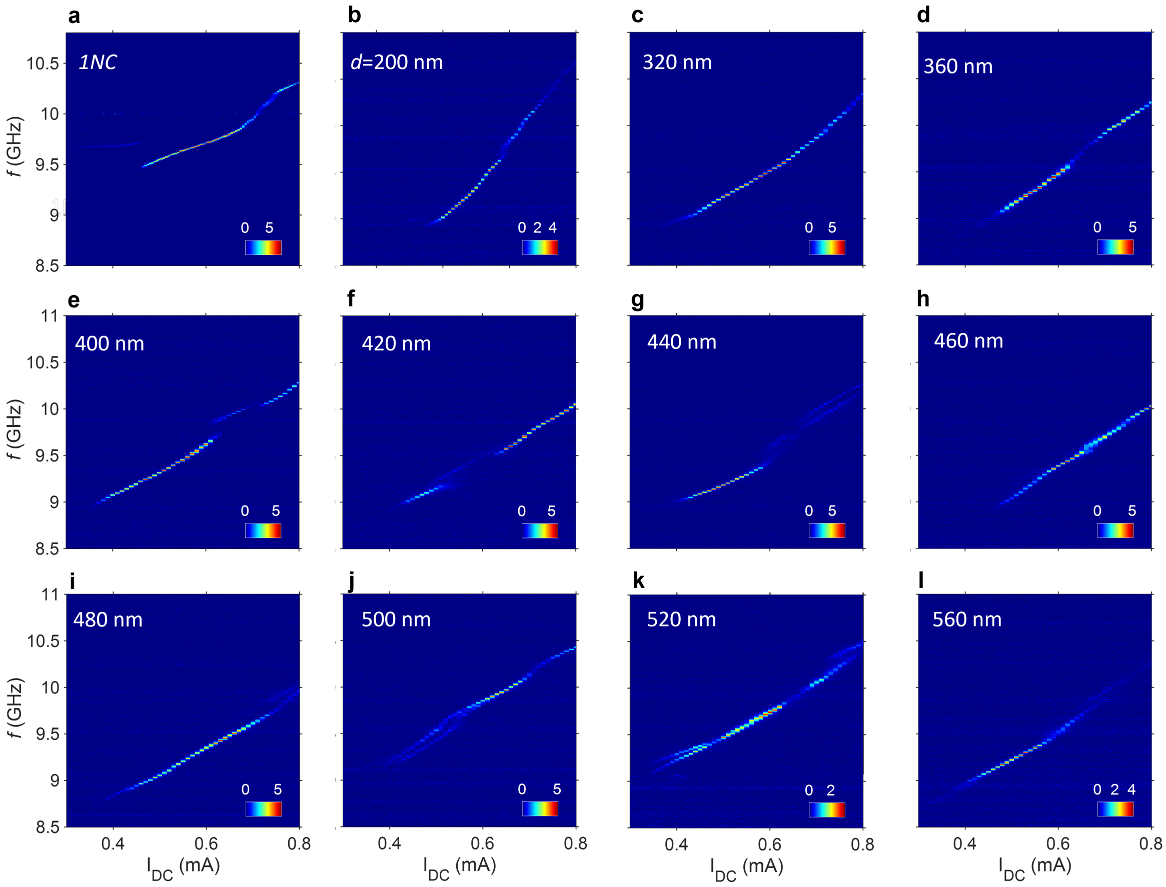

Figure S2: (a-l): PSD *vs.* direct current for single (1NC) and double nano-constriction SHNOs of W/CoFeB/MgO with varying d (= 200-560 nm).

1.3 Supplementary Note 3. Variable phase synchronization at large separations

As we increase the separation of SHNOs, the bridge size (the rhombic area) gets bigger and bigger, resulting in larger damping losses to the spin waves. Hence, to achieve long range spin wave driven mutual synchronization we have to fabricate SHNOs with narrow bridges, to reduce the magnetic area. Using this approach we demonstrate phase controlled mutual synchronization of SHNOs separated by up to 2 μm. Figure S3(a-c) shows the device sketch and observed PSD for double SHNOs separated by 0.7 μm, 1 μm and 2 μm, respectively. These SHNOs also demonstrate a controlled in-phase and out-of-phase synchronization patterns demonstrating long range phase dynamics in these oscillators networks.

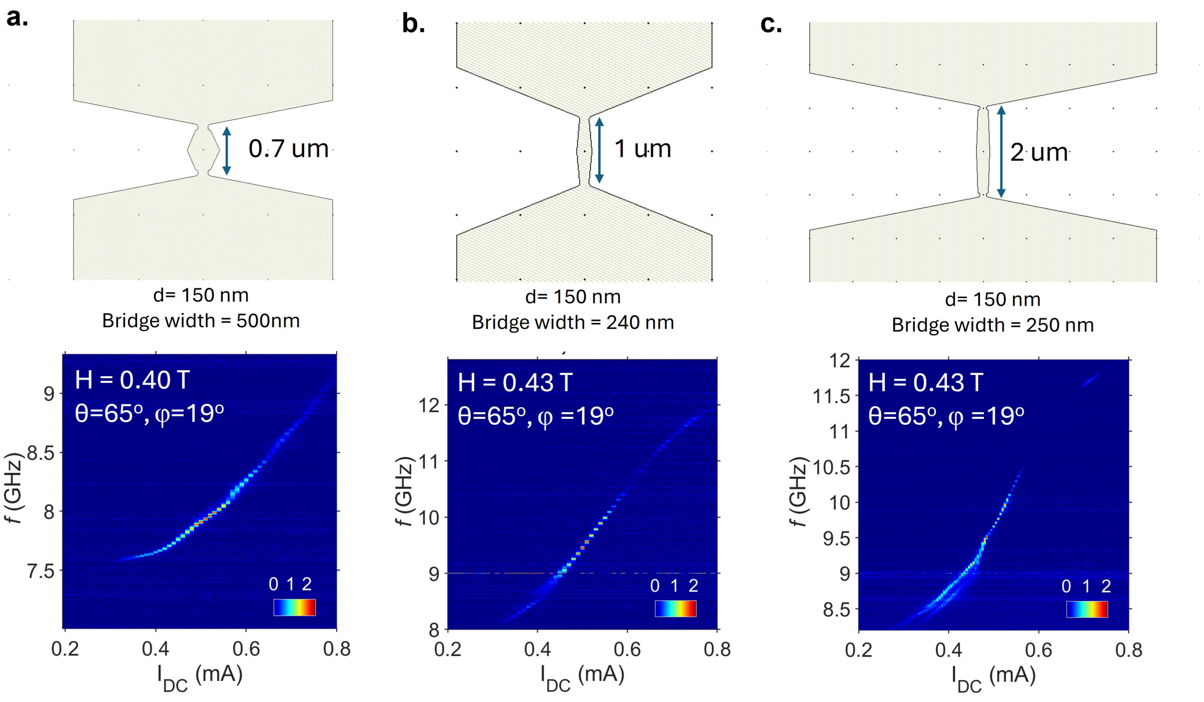

Figure S3: Device schematic and PSD plots for Double SHNOs with (a) d = 150 nm and 500 nm wide bridge, (b) d = 1 μ m and 240 nm wide bridge and (c) d = 2 μ m and 250 nm wide bridge.

1.4 Supplementary Note 4. Magnetic field dependence of variable synchronization

Figure S4(a-l) depict the PSD *vs.* direct current measured for varying field strengths (ranging from 0.35 T – 0.46 T with a step size of 0.01 T) for double nano-constriction SHNOs separated by d = 420 nm. One can clearly see a varying change in the strength of mutual synchronization in these measurements. A noticeable variation in the strength of mutual synchronization is evident in these measurements. Figure S4m illustrates the operating frequency extracted from the PSD plots, revealing that the out-of-phase synchronization regime varies among oscillators and is most pronounced around 0.4 T. This emphasizes the influence of the applied magnetic field on the control of relative phase. However, it is intriguing to observe that the position of out-of-phase locking in terms of direct current does not exhibit significant variation with the magnetic field. This indicates a consistent shift in the spin-wave (SW) gap compared to the ferromagnetic resonance (FMR) gap with the increasing magnetic field, as discussed in the main manuscript.

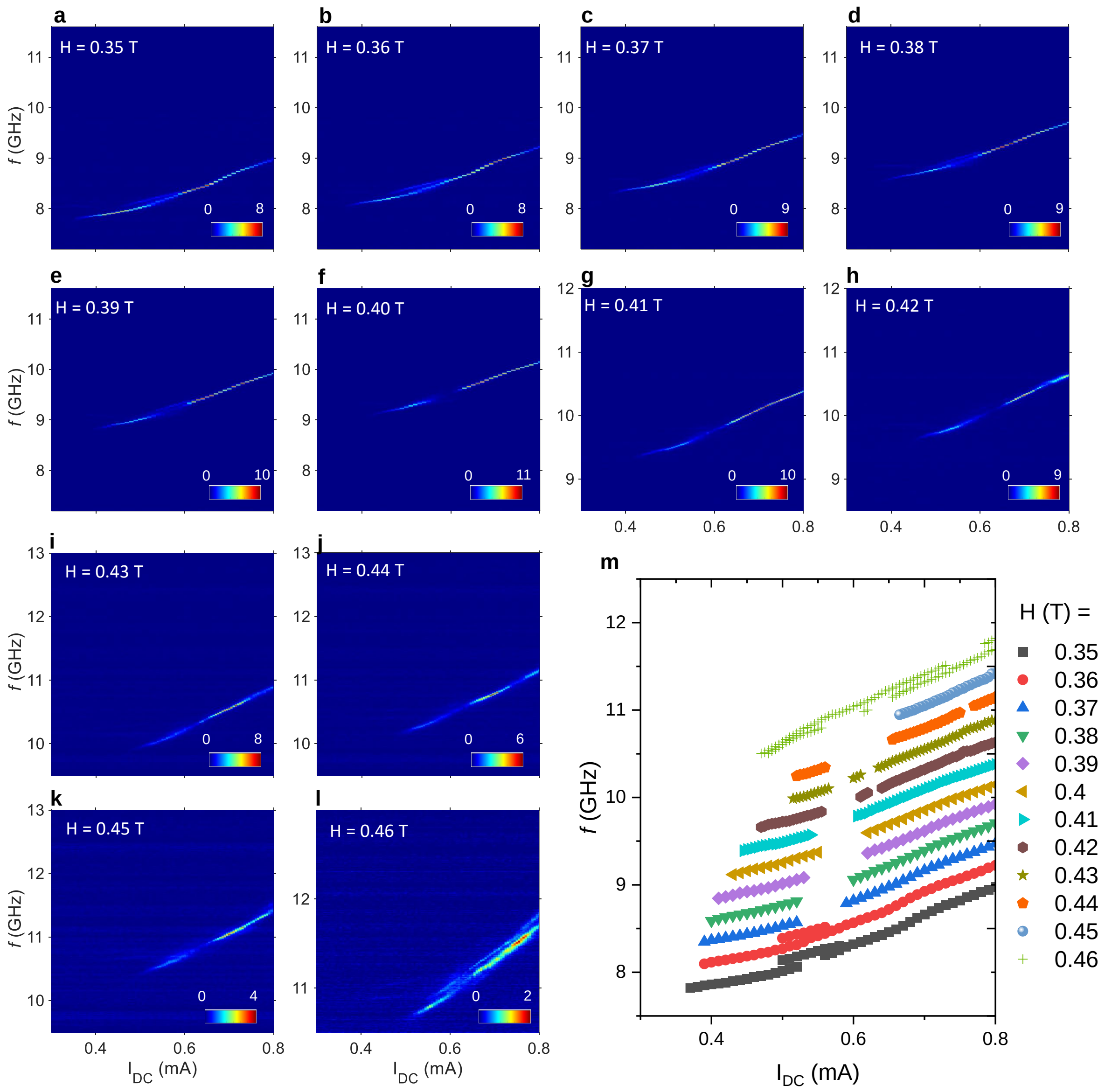

Figure S4: (a-l): PSD *vs.* direct current of double nano-constriction SHNO (W/CoFeB/MgO) with d= 420 nm for varying magnetic field strength (ranging from 0.35 T – 0.46 T with a step size of 0.01 T). All measurements are performed for θ = 65° and φ = 22°. (m): Summarized frequency *vs.* direct current for varying magnetic field.

1.5 Supplementary Note 5. Magnetic field orientation of variable synchronization

Figure S5 shows the dependence of PSD *vs.* direct current on the out-of-plane angle (θ) for double nano-constriction SHNO with d = 420 nm. We observe a complex dependence where for lower OOP angle (53°-60°), we observe

two region III in the PSD plots which starts to merge around 61° and shows single region of out-of phase synchronization until 67°, where we observe two individual modes due to unsynchronization of SHNOs. The complex evolution of mutual synchronization with θ is not clearly understood and would require further detailed analysis.

2 Micro-focused BLS microscopy

2.1 Supplementary Note 6. The relative phase difference between two SHNOs measured at $I_{DC} = 0.70$ mA for different RF power (P_{IL})

To justify the applied P_{IL} = to be -10 dBm, we have measured the phase relation at various P_{IL} for $I_{DC} = 0.70$ mA (out-of-phase synchronization case) as shown in Fig. S6. It can be seen that even -3 dBm RF power is not enough to forcibly drive both oscillators in in-phase synchronization. Hence, the choice of P_{IL} to be -10 dBm seems convincing for our study.

2.2 Supplementary Note 7. μ-BLS microscopy of the individual nano-constrictions with d = 700 nm

Figure S7a shows the PSD *vs.* current measured electrically for the two nano-constrictions separated by d = 700 nm. Fig. S7b shows the full current-dependent spectral distribution of the auto-oscillations, displaying both important similarities and differences when compared with the electrical data shown in Fig. S7a. We observe two faint signals at low currents with about the same threshold. At about 0.35 mA, the two signals merge and the BLS counts increase strongly and remain reasonably high for all higher currents. This rules out the possibility of oscillation death due to the feeble electrical signal consistent with the d = 500 nm case as shown in Fig. 2 of the main manuscript. We present in Fig. S7c-f hybrid frequency-spatial BLS maps for a few selected I_{DC} along a line through the double-nano-constrictions. At $I_{DC} = 0.50$ mA, the spatial maps indicate a synchronized state, with SHNO-1 and SHNO-2 having almost equal BLS counts. At $I_{DC} = 0.525$ mA, the two oscillators are still synchronizing but SHNO-1 now has higher counts than SHNO-2. However, at $I_{DC} = 0.585$ mA, the BLS map looks slightly asymmetrical about its central frequency with unequal counts. Hence, the present result with d = 700 nm (in addition to Fig.2 in the main manuscript) also rules out oscillation death as a possibility and corroborates out-of-phase mutual synchronization as the more likely explanation.

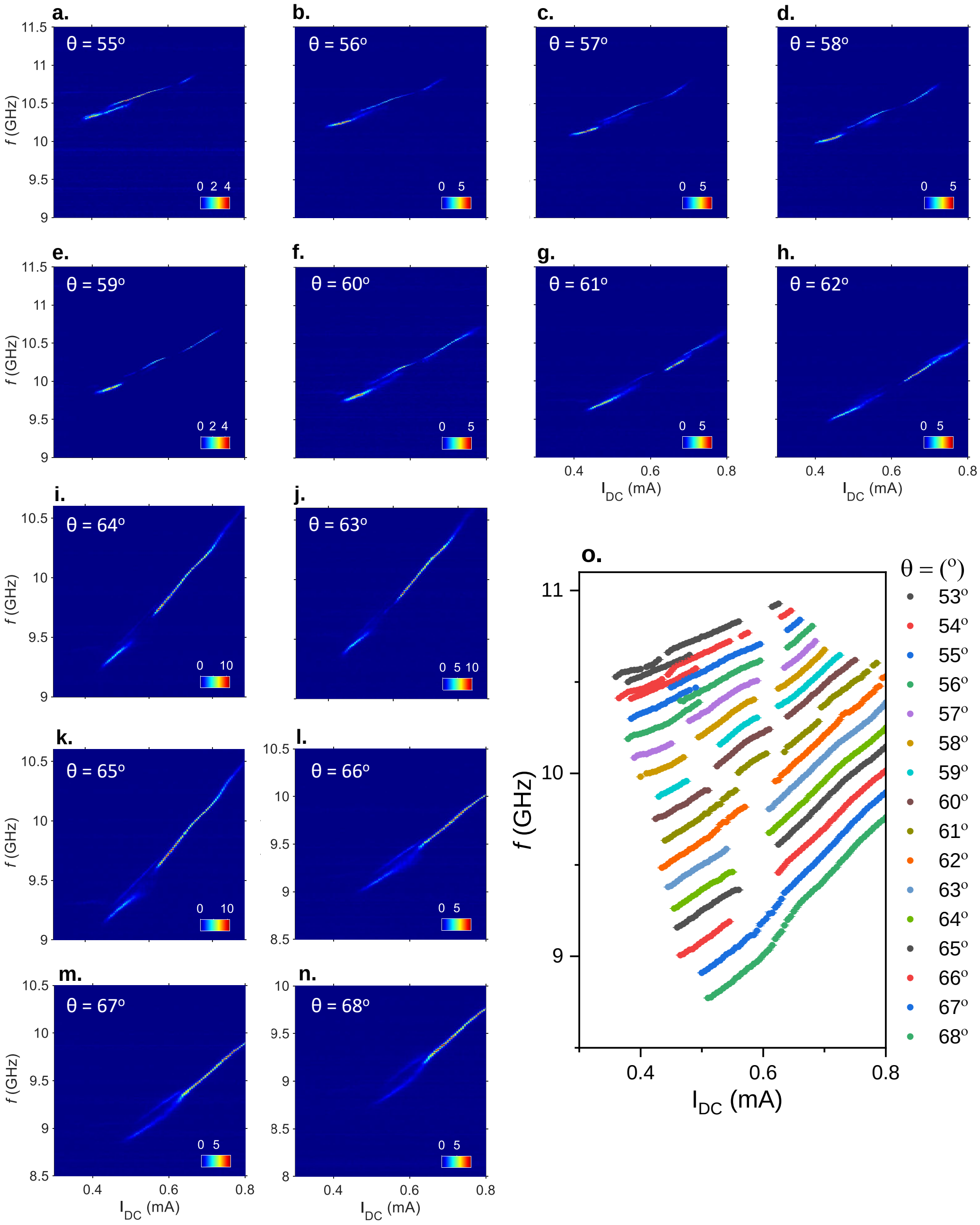

Figure S5: (a-n): PSD *vs.* direct current for varying out-of-plane angle ($\theta = 55^\circ - 68^\circ$) in W/CoFeB/MgO thin film based double nano-constriction SHNO with d= 420 nm. (o.) Summarized extracted frequency *vs.* direct current for PSD plots shown in (a-n).

2.3 Supplementary Note 8. *phase-resolved* μ-BLS microscopy of the individual nano-constrictions with *d* = 700 nm

Figure S8 shows the *phase-resolved* μ-BLS results from the W/CoFeB/MgO device with d = 700 nm at three different current values. At all times, the device is injection-locked with minimal power P_{IL} = –10 dBm and $f_{IL} = f_{SHNO}$. Figure S8a shows a hybrid frequency-spatial map of the phase-BLS counts as a function of frequency and position along the line connecting the two nano-constrictions. Here, the phase angle *w.r.t.* the reference is set to $\phi = 90°$. Figure S8b shows the corresponding counts when the phase shifter is rotated to $\phi = 270°$. It is clear from these two plots that the two nano-constrictions are in phase with each other and contribute about equal counts to the BLS intensity. Fig. S8c shows the full phase dependent BLS counts measured at the location of both constrictions (dashed white lines) when ϕ is varied from 0° to 360°. Sinusoidal fits to the experimental data yield a relative phase difference of $\Delta\phi = 54 \pm 5°$ between the two SHNOs. The phase-resolved μ-BLS results corroborate the electrical measurements' conclusion that the two nano-constrictions largely auto-oscillate in phase. Figures S8d-i show the corresponding phase-dependent results at $I_{DC} = 0.525$ & 0.585 mA. The two nano-constrictions show very different behavior in the BLS maps. When the full phase-dependent counts are fitted, we extract sub-

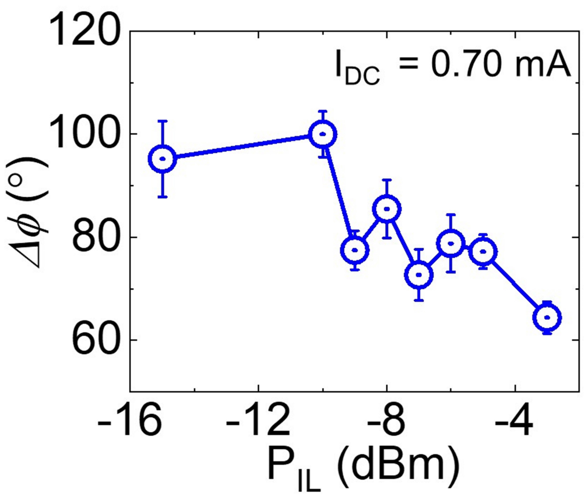

Figure S6: The variation of relative phase difference as a function of P_{IL}. The solid line is just a guide to the eye. The error bar in $\Delta\phi$ is shown by considering the error from the sinusoidal fit of the experimental data *e.g.* Phase BLS counts *vs.* ϕ.

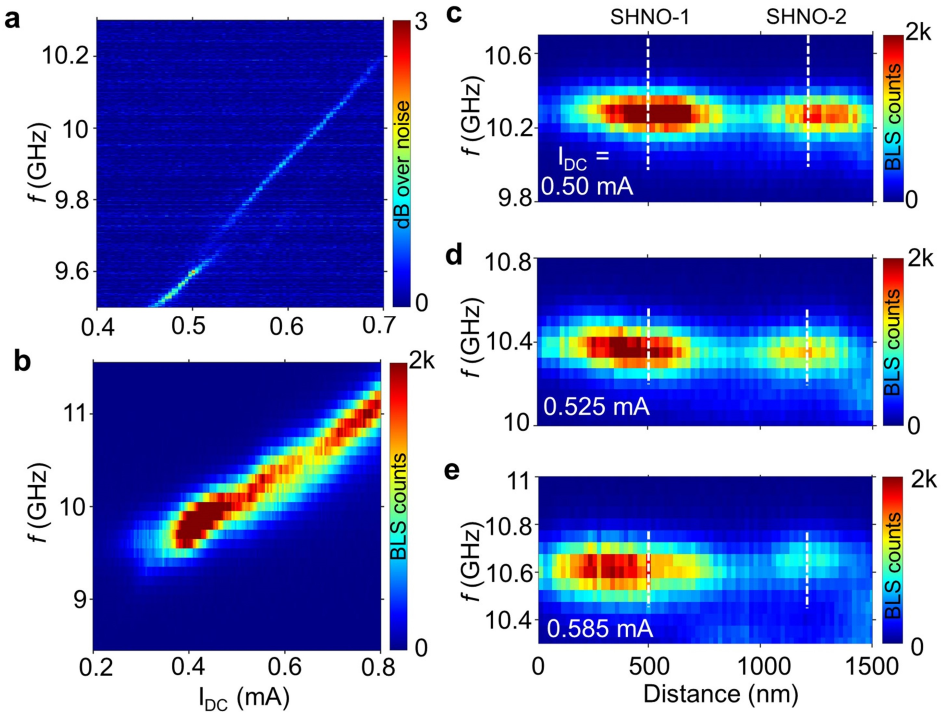

Figure S7: **Spatial mapping using μ-BLS**: (a) Power spectral density *vs.* direct current of double nano-constriction SHNO (W/CoFeB/MgO) with *d*= 700 nm (b) Current-dependent auto-oscillation signal measured using μ-BLS. (c-e) Spin-wave intensity profiles of the double SHNOs along the constrictions, measured at applied current $I_{DC} = 0.50$, 0.525, and 0.585 mA, respectively. The dotted lines refer to the position of the constrictions.

stantial relative phases of $\Delta\phi = 153\pm7°$ at 0.525 mA (correlating the vanishing electrical signal) and $\Delta\phi = 26\pm11°$ at 0.585 mA. Our demonstration of the underlying phenomena of out-of-phase mutual synchronization through phase-resolved BLS measurements for both *d* = 500 nm (main manuscript) and 700 nm-based SHNO devices strongly supports our claim.

3 Micromagnetic simulations

3.1 Supplementary Note 9. Individual oscillators

To completely characterize the dynamics of the system, we extracted the magnetization precession of individual constrictions as a function of time. The spectral modes of the devices were obtained via FFT and are shown in Fig. S9a. We observe that both constrictions auto-oscillate at all current values above the threshold, thus showing

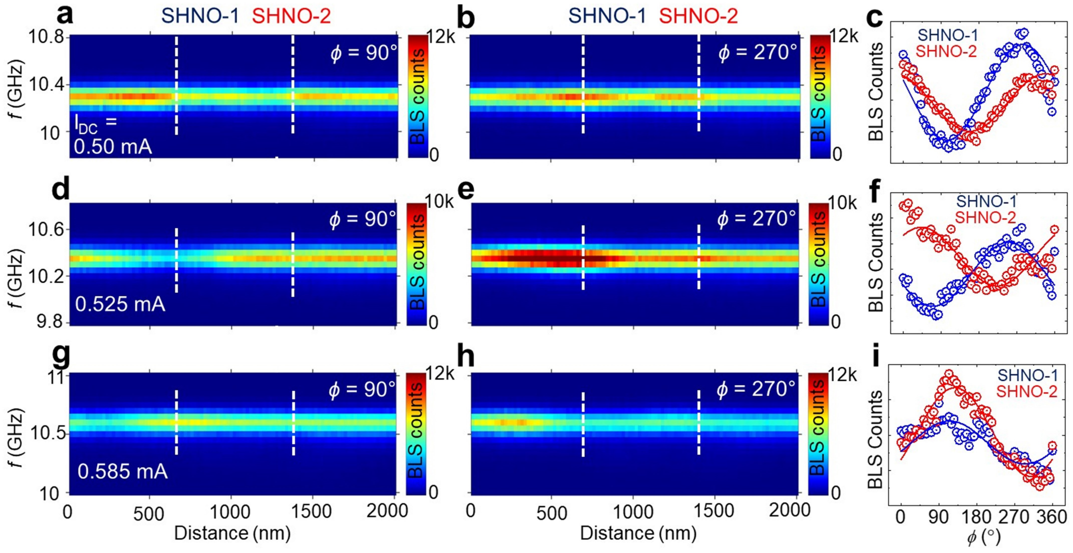

Figure S8: **Phase-resolved μ-BLS measurements:** *Phase-resolved* spin-wave intensity maps of the double SHNOs measured at I_{DC} = 0.50 (a, b), 0.525 (d, e) and 0.585 mA (g, h), with two different phase settings (ϕ) separated by 180°. The last column (c, f, i) shows the BLS counts as a function of ϕ measured at the center of each nano-constriction. The symbols are the measured counts at an injection of P_{IL} = -10 dBm; the solid lines are sinusoidal fits.

that there is no oscillation death, differing from previously reported results in spin torque nano-oscillators (See Ref. 49, main manuscript). We can observe that the constrictions never cease to oscillate and the apparent amplitude death is due to a broadening of the linewidth of each individual oscillator as they pull on each other's frequency. This broadening lowers the overall device power below the noise floor of the measurement and thus the apparent amplitude death.

Further evidence of continuous oscillations is shown in **Video 1** (additional supplemental material). In this animation, we show the steady state oscillation of the device at the anti-phase (425 μA) and in-phase (500 μA) currents.

3.2 Supplementary Note 10. Phase difference between oscillators

We obtained the phase differences between the oscillators as a function of the applied direct current shown in Fig. 5 (main manuscript), by averaging the phase calculated from the complex part of the point-resolved FFT maps. Specifically, we extracted the phase along the green line in Fig. S9b of the device and subtracted one from the

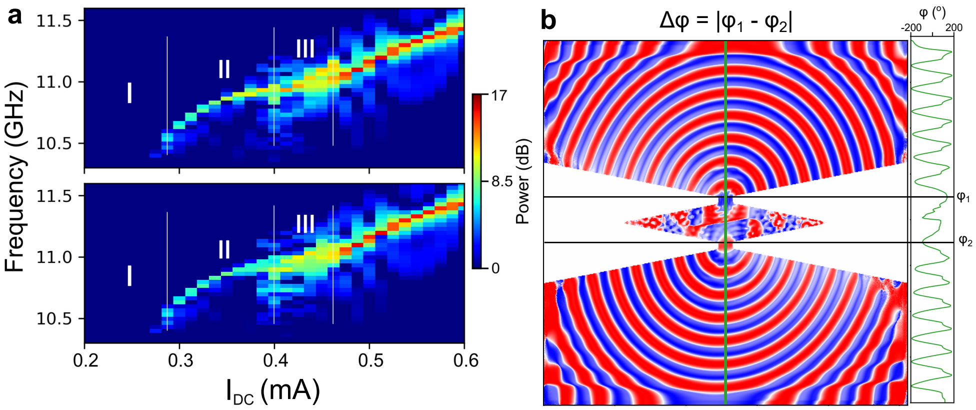

Figure S9: (**a**) PSDs of individual oscillators. The anti-phase synchronization in regime III causes a broadening of the linewidth of the oscillators and a reduction of the overall device power. (**b**) Procedure for calculating the phase difference $\Delta\phi$ between the nano-constrictions. The phase along the green line is shown in the right.

other.

3.3 Supplementary Note 11. Wave-length of spin waves versus I_{DC}

Figure S10 shows the wavelength of spin waves versus charge current (I_{DC}) calculated from the simulated intensity maps of micromagnetic simulation presented in the main manuscript and previous figure. The continuous change in wavelength arises from the change in operating frequency of the oscillators.

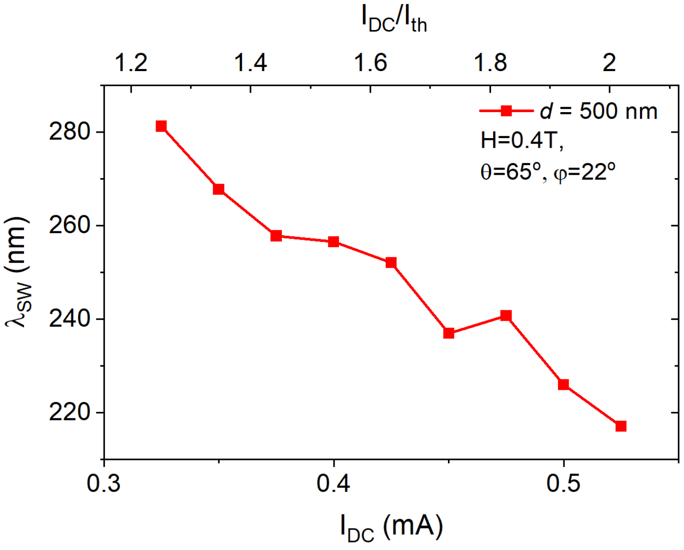

Figure S10: Wavelength of propagating spin waves outside the nano-constriction region in two mutual synchronized SHNOs with separation d = 500 nm.
