# Supplementary figures and images for: Spin-wave-mediated mutual synchronization and phase tuning in spin Hall nano-oscillators
Source: Nat Phys. 2025 Jan 8;21(2):245–52. doi: 10.1038/s41567-024-02728-1 (PMC11825361; doi:10.1038/s41567-024-02728-1)

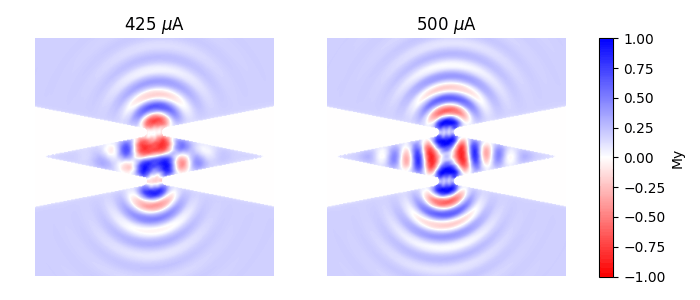

Supplement: Supplementary file 2 — Micromagnetic simulation of PSWs in SHNOs at 425 μA and 500 μA charge current. [file 41567_2024_2728_MOESM2_ESM.gif]
